# Complete Genome Sequence of a Hop Latent Virus Infecting Hop Plants

**DOI:** 10.1128/genomeA.00302-15

**Published:** 2015-04-23

**Authors:** Yeonhwa Jo, Hoseong Choi, Won Kyong Cho

**Affiliations:** Department of Agricultural Biotechnology, College of Agriculture and Life Sciences, Seoul National University, Seoul, Republic of Korea

## Abstract

The hop latent virus is a single-stranded RNA virus that mainly infects hop plants. Here, we report the complete genome sequence of a hop latent virus, which was *de novo* assembled by RNA sequencing (RNA-seq). Our study indicates that transcriptome data are useful for identifying a complete viral genome.

## GENOME ANNOUNCEMENT

The members of the genus *Carlavirus* in the family *Betaflexiviridae* are single-stranded positive-sense RNA viruses. Of them, the hop latent virus (HpLV) is one of three carlaviruses that also includes hop mosaic virus and American hop latent virus (1–3). They infect the cones of various hop (*Humulus lupulus* L.) cultivars, which are important bitter flavoring agents used in the production of beer (2, 3). The HpLV-infected hop plants do not show any visible symptoms, and the hop production damage caused by HpLV has not been determined (3). In general, HpLV is transmitted in a nonpersistent manner by both aphids and mechanical inoculation (3). HpLV consists of filamentous RNA particles that encode six proteins (1).

To determine the causal HpLV agents of hops, we searched for publicly available hop transcriptome data (4). Hop samples were collected from four different *Humulus lupulus* L. cultivars, including Taurus, Nugget, Magnum, and Apollo. Three different tissues, including leaves, hop cones without lupulin glands, and lupulin glands, were used for the total RNA extraction. A total of 11 libraries were sequenced by an Illumina HiSeq 2000, and all raw data were subjected to transcriptome assembly using the Trinity program, followed by CD-HIT (version 4.5) to remove redundant transcripts (4). Out of the assembled 174,938 transcripts from previous hop transcriptome data (4), six transcripts were associated with HpLV. Of them, a transcript containing 8,657 nucleotides (nt) was the longest transcript that was homologous to the reference HpLV sequence (accession no. NC_002552.1). The sequence alignment to the reference sequence revealed that the transcript contained an insertion of 22 nucleotides at the 5′ terminus and 23 poly(A) nucleotides at the 3′ terminus. After removing the inserted nucleotides at the 5′ and 3′ termini, a complete HpLV genome sequence with a length of 8,612 nt was obtained. We confirmed that all 11 hop libraries were infected by HpLV, indicating the presence of HpLV infection in four different hop cultivars. We named the newly identified HpLV genome HpLV isolate Taurus (accession no. KP861891). The HpLV isolate Taurus genome contains six open reading frame genes, which encode a viral replicase, three triple gene block proteins, a coat protein, and a cysteine-rich protein. So far, only three genome sequences for HpLV, including isolate Taurus, are available. The sizes of all three genomes are identical. The isolate Taurus is closely related to the HpLV isolate Zatec 2008, which was identified from hops from Poland (accession no. HG793797.1) (5). Taken together, our study reports the successful application of transcriptome data to identify a complete genome sequence of HpLV isolate Taurus.

### Nucleotide sequence accession number. 

The genome sequence of hop latent virus isolate Taurus has been submitted to GenBank under the accession no. KP861891.

## References

[1] Hataya T, Uchino K, Arimoto R, Suda N, Sano T, Shikata E, Uyeda I 2000 Molecular characterization of hop latent virus and phylogenetic relationships among viruses closely related to carlaviruses. Arch Virol 145:2503–2524. doi:10.1007/s007050070005. 11205102

[2] Hataya T, Arimoto R, Suda N, Uyeda I 2001 Molecular characterization of *Hop mosaic virus*: its serological and molecular relationships to *Hop latent virus*. Arch Virol 146:1935–1948. doi:10.1007/s007050170043. 11722015

[3] Adams AN, Barbara DJ 1982 Host range, purification and some properties of two carlaviruses from hop (*Humulus lupulus*): hop latent and American hop latent. Ann Appl Biol 101:483–494. doi:10.1111/j.1744-7348.1982.tb00849.x.

[4] Clark SM, Vaitheeswaran V, Ambrose SJ, Purves RW, Page JE 2013 Transcriptome analysis of bitter acid biosynthesis and precursor pathways in hop (*Humulus lupulus*). BMC Plant Biol 13:12. doi:10.1186/1471-2229-13-12. 23347725 PMC3564914

[5] Ziegler A, Kawka M, Przybys M, Doroszewska T, Skomra U, Kastirr U, Matoušek J, Schubert J 2014 Detection and molecular analysis of *Hop latent virus* and *Hop latent viroid* in hop samples from Poland. J Cult Plants 66:248–254.

